# RETROSPECTIVE ANALYSIS OF MORTALITY DETERMINANTS IN COVID-19 PATIENTS BASED ON CLINICAL DIAGNOSES PRIOR TO DEATH AT A NATIONAL REFERRAL HOSPITAL IN INDONESIA

**DOI:** 10.21010/Ajidv19i2S.3

**Published:** 2025-10-17

**Authors:** SUSANTI Rika, AFRIANI Afriani

**Affiliations:** 1 Department of Forensic and Medicolegal, Faculty of Medicine, Universitas Andalas, Padang, Indonesia, 25127; 2 Department of Pulmonology and Respiratory Medicine, Dr. M. Djamil General Hospital, Padang, Indonesia

**Keywords:** COVID-19, mortality, co-occurring clinical conditions, acute respiratory distress syndrome

## Abstract

**Background:**

Mortality in COVID-19 patients is often the result of multiple overlapping clinical complications rather than a single cause. Understanding these patterns is essential for improving clinical outcomes and supporting accurate forensic evaluation.

**Aim:**

This study aimed to identify the clinical conditions contributing to death in COVID-19 patients and to assess the association between the number of co-occurring conditions and patient outcomes.

**Methods:**

A retrospective observational study was conducted on 100 confirmed COVID-19 patients admitted to a national referral hospital in Indonesia. Data on demographic characteristics, comorbidities, clinical severity, and outcomes were collected from medical records. Clinical causes of death were recorded and categorized by the number of co-occurring conditions. Unadjusted and adjusted odds ratios (ORs) for mortality were calculated using logistic regression, adjusting for age, sex, BMI, comorbidities, and disease severity.

**Results:**

The most frequently documented clinical conditions contributing to death were acute respiratory distress syndrome (68%), respiratory failure (55%), and septic shock (34%). Among patients, 30% had one condition, 45% had two, and 25% had three or more. Compared to patients with a single condition, those with two conditions had an adjusted OR of 2.41 (95% CI: 1.16–4.41), and those with three or more conditions had an adjusted OR of 19.4 (95% CI: 10.73–32.23).

**Conclusion:**

Mortality in COVID-19 is significantly associated with the number of co-occurring clinical conditions. Early detection and integrated management of overlapping complications are essential for reducing fatal outcomes and improving diagnostic accuracy in clinical and forensic settings.

## Introduction

Since the emergence of the novel coronavirus disease (COVID-19) in late 2019, the world has witnessed an unprecedented global health crisis (Pollard *et al.*, 2020). Caused by the severe acute respiratory syndrome coronavirus 2 (SARS-CoV-2), COVID-19 has led to millions of deaths worldwide and has placed immense pressure on healthcare systems, particularly in low- and middle-income countries (Muralidar *et al.*, 2020). While most COVID-19 patients experience mild to moderate symptoms, a significant proportion develop severe illness requiring hospitalization and critical care, and in many cases, resulting in death (Lai *et al.*, 2020). Understanding the clinical features associated with COVID-19-related mortality is crucial for improving early recognition, timely intervention, and appropriate clinical management (Sharma *et al.*, 2020).

Numerous studies across various countries have examined the clinical causes of death among COVID-19 patients (Ramanova *et al.*, 2021; Estenssoro *et al.*, 2022). The most commonly reported condition is acute respiratory distress syndrome (ARDS), followed by sepsis, multi-organ failure, and cardiovascular complications such as acute myocardial infarction or pulmonary embolism (Meyer *et al.*, 2021). These clinical conditions often present with symptoms such as severe dyspnea, hypoxemia, hypotension, altered consciousness, and evidence of systemic inflammation. Accurate identification of these fatal complications through clinical evaluation is essential to determine disease severity and guide treatment decisions (Filip *et al.*, 2022).

Despite the growing body of literature on COVID-19 mortality, several limitations and gaps persist. Much of the available evidence originates from high-income countries in North America, Europe, and China (Boro *et al.*, 2022; Filip *et al.*, 2022). These studies may not adequately represent the healthcare infrastructure, resource availability, and patient characteristics in low- and middle-income countries such as Indonesia (Estenssoro *et al.*, 2022; Filip *et al.*, 2022). Moreover, few studies have focused exclusively on clinical diagnostic indicators of death, especially in tertiary referral hospitals where more severe cases are concentrated (Meyer *et al.*, 2021; Estenssoro *et al.*, 2022). This lack of context-specific data limits the ability to generalize findings and hinders the development of appropriate clinical response strategies in similar settings.

Indonesia, as one of the Southeast Asian countries most affected by the COVID-19 pandemic, offers a critical context for examining the clinical pathways that lead to death (Hartantri *et al.*, 2023; Harapan *et al.*, 2023). National referral hospitals in Indonesia have functioned as key centers for treating severe and critical COVID-19 cases (Harapan *et al.*, 2023). Evaluating the clinical conditions that directly contribute to mortality in these settings provides vital insights into patient outcomes and can inform future preparedness and clinical response plans (Rampal *et al.*, 2021).

This study aims to address existing gaps by analyzing the clinical characteristics contributing to death among COVID-19 patients treated at a national referral hospital in Indonesia. The findings are expected to contribute to a better understanding of the progression and fatal outcomes of COVID-19, particularly in resource-limited hospital settings, and to support the development of targeted interventions to reduce avoidable deaths during ongoing and future pandemics.

## Materials and Methods

### Study Design and Setting

This study employed a descriptive retrospective design conducted at Dr. M. Djamil General Hospital, a national referral hospital located in Padang, Indonesia. As one of the primary referral centers for COVID-19 management in the region. The study reviewed medical records of deceased COVID-19 patients admitted between March 2020 and December 2022.

### Study Participants and Sample Size

The study population consisted of all confirmed COVID-19 patients who were hospitalized during the study period. Patients were included if they had a laboratory-confirmed diagnosis of COVID-19 via RT-PCR and complete documentation of clinical assessments. This documentation included a detailed record of clinical evaluations, medical treatments administered, and any relevant clinical observations made during their hospital stay. Patients with incomplete records, defined as missing key clinical information such as clinical progress notes or treatment regimens, or those whose deaths were due to trauma or causes unrelated to COVID-19 (e.g., non-viral infections or chronic conditions such as cancer), were excluded. Using convenience sampling, all eligible cases that met the criteria during the specified period were included, resulting in 100 confirmed COVID-19 patients.

### Sampling Techniques

A convenience sampling method was applied to include all confirmed COVID-19 patients who met the inclusion criteria during the study period. This approach was chosen due to the specific and limited number of cases available within the defined time frame, allowing for comprehensive inclusion without randomization bias. All eligible cases that met the criteria were included in the study.

### Operational Definition

In this study, a confirmed case of COVID-19 was defined as any patient who tested positive for SARS-CoV-2 using real-time polymerase chain reaction (RT-PCR) from a nasopharyngeal swab specimen (Moreira *et al.*, 2021). The cause of death was determined based on documentation in the patient’s medical records, as concluded by the attending physician, and supported by clinical findings performed prior to death. This approach aligns with the World Health Organization’s guidelines for certifying COVID-19 as a cause of death, which emphasize the importance of recording the causal sequence leading to death in the medical certificate of cause of death (World Health Organization, 2020).

Clinical diagnoses leading to death included several critical conditions commonly associated with severe COVID-19 progression. These included ARDS, defined by acute onset of respiratory failure with bilateral lung infiltrates and hypoxemia unresponsive to oxygen therapy; respiratory failure, indicated by inadequate gas exchange requiring ventilatory support; cardiac arrest, characterized by the sudden cessation of effective cardiac activity; septic shock, identified by persistent hypotension despite adequate fluid resuscitation and signs of tissue hypoperfusion; disseminated intravascular coagulation (DIC), marked by systemic activation of blood coagulation leading to microvascular thrombosis and hemorrhagic complications; and massive pulmonary embolism, suspected clinically and/or confirmed by imaging as large thromboemboli obstructing the pulmonary arteries (Matthay *et al.*, 2015; Moreira *et al.*, 2021; Swenson *et al.*, 2021).

### Data Collection Technique

Data were collected through a review of electronic and physical medical records by trained research assistants. The research assistants underwent a comprehensive training program, which included detailed instructions on the proper handling of medical records, standardized procedures for data extraction, and guidance on how to accurately interpret clinical information. The training also covered confidentiality protocols to ensure patient privacy and accuracy in data collection. A standardized data extraction form was used to collect demographic characteristics, comorbidities, clinical symptoms, and documented cause of death.

### Ethical Considerations

This study was approved by the Ethics Committee of Dr. M. Djamil General Hospital, Padang, Indonesia under protocol number DP.04.03/D.XVI.IX/412/2023. All data were anonymized and handled in accordance with institutional and national ethical standards to protect patient confidentiality. As this was a retrospective chart review, the requirement for informed consent was waived.

## Data Analysis

Descriptive statistics were used to summarize patient demographics, clinical features, and radiological findings. Categorical variables were presented as frequencies and percentages, while continuous variables were described using medians and interquartile ranges, as appropriate. Cross-tabulation was used to explore the correlation between clinical and radiological findings in relation to the cause of death. Data analysis was conducted using SPSS software.

## Results

Characteristics of the study participants (Table 1).

**Table 1:** Characteristics of the study participants

Variables	f(%)	Median (IQR)
**Sex, f(%)**		
Male	55 (55.0)	
Female	45 (45.0)	
**Age (years)**		62 (54-72)
**Body mass index (BMI), f(%)**		
Underweight (<18.5 kg/m^2^)	17 (17.0)	
Normal (18.5-23.49 kg/m^2^)	70 (70.0)	
Overweight (23.5-24.99 kg/m^2^)	13 (13.0)	
**Comorbidities**		
Hypertension	50 (50.0)	
Diabetes mellitus	40 (40.0)	
Chronic kidney disease	2 (2.0)	
Coronary artery disease	8 (8.0)	
**Length of stay (days)**		11 (3-19)
**Severity, f(%)**		
Severe	21 (21.0)	
Moderate	70 (70.0)	
Mild	9 (9.0)	
**Outcome, f(%)**		
Life	58 (58.0)	
Death	42 (42.0)	

Table 1 summarizes the demographic and clinical characteristics of the 100 study participants. The distribution of sex was relatively balanced, with 55% males and 45% females. The median age was 62 years, with an interquartile range (IQR) of 54 to 72 years. Regarding nutritional status, based on BMI, 70% of participants had a normal BMI, while 17% were underweight and 13% were classified as overweight. In terms of comorbidities, hypertension was present in 50% of participants, followed by diabetes mellitus in 40%, coronary artery disease in 8%, and chronic kidney disease in 2% of the sample.

The median of hospital stay was 11 days, with a range of 3 to 19 days. The majority of patients experienced moderate disease severity (70%), while 21% were classified as severe cases and 9% as mild. As for outcomes, 58% of participants survived, whereas 42% died during the study period.

Documented clinical conditions contributing to death in COVID-19 patients (Table 2).

**Table 2:** Documented clinical conditions contributing to death in COVID-19 patients

Variables	f(%)
Cause of death*	
Acute respiratory distress syndrome (ARDS)	68 (68.0)
Respiratory failure	55 (55.0)
Septic shock	34 (34.0)
Cardiac arrest	29 (29.0)
Disseminated intravascular coagulation (DIC)	17 (17.0)
Massive pulmonary embolism	12 (12.0)
**Co-occurring clinical conditions prior to death**	
1 condition	30 (30.0)
2 conditions	45 (45.0)
3 or more conditions	25 (25.0)

* , Patients may have more than one clinical condition contributing to death

Table 2 presents the documented clinical conditions that contributed to the deaths of COVID-19 patients. The most frequently observed condition was acute respiratory distress syndrome (ARDS), reported in 68 patients (68.0%). This was followed by respiratory failure in 55 patients (55.0%) and septic shock in 34 patients (34.0%). Additionally, cardiac arrest was noted as a terminal event in 29 patients (29.0%), while disseminated intravascular coagulation (DIC) and massive pulmonary embolism were documented in 17 (17.0%) and 12 (12.0%) patients, respectively. It is important to note that many patients experienced more than one life-threatening complication. Regarding the extent of overlapping conditions, 30 patients (30.0%) had a single clinical condition contributing to death, 45 patients (45.0%) had two co-occurring conditions, and 25 patients (25.0%) had three or more.

**Figure 1:**
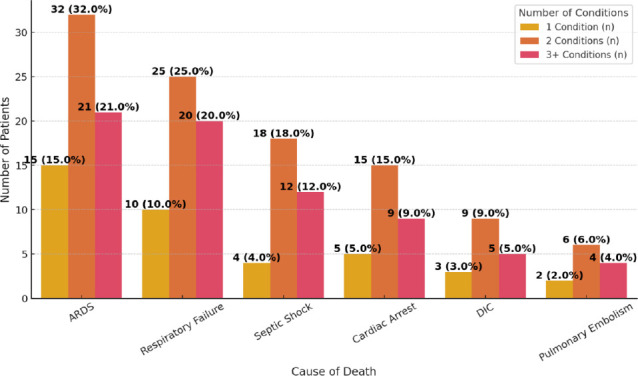
Distribution of co-occurring clinical conditions for each cause of death in COVID-19 patients

Distribution of co-occurring clinical conditions for each cause of death in COVID-19 patients (Figure 1).

Figure 1 shows the distribution of co-occurring clinical conditions for each cause of death in COVID-19 patients. ARDS was the most frequent condition, present alone in 15% of patients, with one other condition in 32%, and with multiple conditions in 21%. Respiratory failure followed a similar pattern, with 10%, 25%, and 20% respectively. Septic shock, cardiac arrest, DIC, and pulmonary embolism were more often observed in patients with two or more co-existing conditions. These findings underscore that most fatal cases involved multiple critical conditions, highlighting the complex clinical course of severe COVID-19.

Co-occurring clinical conditions and patient outcome (Table 3).

**Table 3:** Co-occurring clinical conditions and patient outcome

Co-occurring clinical conditions	Death (f/%)	Life (f/%)	Unadjusted OR (95% CI)	Adjusted OR (95% CI)*
1 condition	4 (13.3)	26 (86.7)	Ref	Ref
2 conditions	18 (40.0)	27 (60.0)	4.33 (1.29-14.53)	2.41 (1.16-4.41)
3 or more conditions	20 (80.0)	5 (20.0)	26.0 (6.17-109.54)	19.4 (10.73-32.23)

* Adjusted for sex, age, BMI, comorbidities, and severity

Table 3 presents the relationship between the number of co-occurring clinical conditions and patient outcomes in COVID-19 cases. After adjusting for potential confounding variables, including sex, age, BMI, comorbidities, and disease severity, the association remained statistically significant. Patients with two conditions had an adjusted odds ratio (AOR) of 2.41 (95% CI: 1.16–4.41), while those with three or more conditions had an AOR of 19.4 (95% CI: 10.73–32.23).

## Discussion

This study provides a comprehensive analysis of the clinical conditions contributing to mortality among hospitalized COVID-19 patients in a national referral hospital in Indonesia. The findings reveal that mortality in COVID-19 is often the result of multiple co-occurring clinical complications, most notably ARDS, respiratory failure, septic shock, and thromboembolic events. The results demonstrate a strong dose-response relationship: the risk of death significantly increases with the number of overlapping clinical conditions, even after adjustment for age, sex, BMI, comorbidities, and disease severity.

Our findings are consistent with a growing body of global evidence that ARDS and multi-organ dysfunction are the primary clinical pathways leading to COVID-19 mortality (Pformueller *et al.*, 2021; Dong *et al.*, 2023). Previous study reported ARDS in over 90% of non-survivors, frequently accompanied by sepsis and cardiac complications (Wu *et al.*, 2020). Similarly, another study found that multi-system organ failure was more predictive of poor outcomes than individual comorbidities alone (Torres Acosta *et al.*, 2020).

The contribution of overlapping conditions to mortality risk, as shown in this study, reinforces previous observations, who emphasized the compounding effects of systemic inflammation and coagulopathy in ICU patients with COVID-19 (Grasselli *et al.*, 2020; Nindrea *et al.*, 2020). By quantifying the odds of death based on the number of clinical complications, our findings add to the understanding of how clinical deterioration occurs in a cascading fashion (Gorlinger *et al.*, 2020).

This study builds upon existing knowledge by providing locally contextualized data from a low- to middle-income country setting, which has been underrepresented in the literature. The adjusted odds ratios presented here demonstrate that even after accounting for common risk factors, the number of concurrent conditions remains a powerful and independent predictor of death (Cheshire *et al.*, 2021; Esposito *et al.*, 2022; Nindrea *et al.*, 2023). This has important implications for clinical triage and risk stratification protocols in resource-limited hospitals.

Furthermore, our analysis of co-occurring conditions by cause of death offers granular insight into the complexity of fatal COVID-19 cases. The majority of patients did not die from a single complication but from a combination of pathologies emphasizing the need for multidisciplinary and dynamic clinical management strategies.

While our findings align with most global studies, there are discrepancies regarding the role of age. Interestingly, in our adjusted analysis, age ≥60 was not independently associated with increased mortality after adjusting for co-occurring conditions and disease severity. This differs from several large-scale studies that found age to be a dominant predictor of death. The discrepancy may be due to sample composition, survival bias, or the relatively high baseline severity in our study cohort, which could have masked the age effect (Matthay *et al.*, 2015; Moreira *et al.*, 2021; Swenson *et al.*, 2021).

Additionally, while comorbidities such as hypertension and diabetes were prevalent among deceased patients, they did not independently predict mortality in our adjusted model, unlike findings from studies in higher-income settings. This may reflect differences in healthcare access, case management, or the threshold for hospitalization in Indonesia (Ayubi et al., 2022; Harapan *et al.*, 2023).

Several limitations should be noted, particularly for application in the forensic field. First, this study is based on retrospective hospital records and may lack standardization in how clinical causes of death were determined or documented. There is potential for misclassification or underreporting of certain fatal conditions, such as myocardial infarction or pulmonary embolism, if not confirmed through imaging or autopsy.

Second, radiological confirmation of causes of death (e.g., PE, DIC) was not integrated into the final analysis, which may limit the forensic accuracy of attributing specific complications. Forensic pathology often relies on postmortem findings to establish the true cause of death, which was not possible in this clinical cohort. Therefore, the generalization of these findings to forensic investigations where definitive cause of death must be determined should be approached with caution.

Finally, the presence of confounding clinical interventions (e.g., mechanical ventilation, anticoagulation therapy) was not accounted for in the regression model, which may influence both the development of complications and survival outcomes.

## Conclusion

This study highlights that mortality in hospitalized COVID-19 patients is strongly associated with the presence of multiple co-occurring clinical conditions, particularly acute respiratory distress syndrome, respiratory failure, and septic shock. The risk of death increases substantially with the number of complications, even after adjusting for age, sex, BMI, comorbidities, and disease severity. These findings underscore the importance of early identification and aggressive management of overlapping clinical conditions in severe COVID-19 cases. In forensic contexts, these results support the need for comprehensive clinical documentation and multidisciplinary evaluation to accurately determine cause of death.

### Conflict of Interest

The authors declares that there is no conflict of interest associated with this study.

**List of Abbreviations:**
COVID-19: Coronavirus Disease 2019;
AOR: Adjusted Odds Ratio;
ARDS: Acute Respiratory Distress Syndrome;
BMI: Body Mass Index;
CI: Confidence Interval;
COVID-19: Coronavirus disease;
DIC: Disseminated Intravascular Coagulation;
ICU: Intensive Care Unit;
OR: Odds Ratio;
SARS-CoV-2: Severe Acute Respiratory Syndrome Coronavirus 2.
